# Lethal and Sublethal Toxicity of Nanosilver and Carbon Nanotube Composites to *Hydra vulgaris*—A Toxicogenomic Approach

**DOI:** 10.3390/nano14231955

**Published:** 2024-12-05

**Authors:** Joelle Auclair, Eva Roubeau-Dumont, François Gagné

**Affiliations:** Environment and Climate Change Canada, Aquatic Contaminant Research Division, 105 McGill, Montreal, QC H1S 1E7, Canada; joelle.auclair@ec.gc.ca (J.A.); eva.roubeaudumont@ec.gc.ca (E.R.-D.)

**Keywords:** *Hydra vulgaris*, sublethal toxicity, oxidative stress, dopamine, autophagy, oxidative stress, regeneration

## Abstract

The increasing use of nanocomposites has raised concerns about the potential environmental impacts, which are less understood than those observed with individual nanomaterials. The purpose of this study was to investigate the toxicity of nanosilver carbon-walled nanotube (AgNP–CWNT) composites in *Hydra vulgaris*. The lethal and sublethal toxicity was determined based on the characteristic morphological changes (retraction/loss of tentacles and body disintegration) for this organism. In addition, a gene expression array was optimized for gene expression analysis for oxidative stress (superoxide dismutase, catalase), regeneration and growth (serum response factor), protein synthesis, oxidized DNA repair, neural activity (dopamine decarboxylase), and the proteasome/autophagy pathways. The hydras were exposed for 96 h to increasing concentrations of single AgNPs, CWNTs, and to 10% AgNPs–90% CWNTs, and 50% AgNPs–50% CNWT composites. Transmission electron microscopy (TEM) and energy dispersive X-ray spectroscopy (EDS) analysis revealed the presence of AgNPs attached to the carbon nanotubes and AgNP aggregates. The data revealed that the AgNP–CWNT composites were more toxic than their counterparts (AgNPs and CNWT). The sublethal morphological changes (EC50) were strongly associated with oxidative stress and protein synthesis while lethal morphological changes (LC50) encompassed changes in dopamine activity, regeneration, and proteasome/autophagic pathways. In conclusion, the toxicity of AgNP–CWNT composites presents a different pattern in gene expression, and at lower threshold concentrations than those obtained for AgNPs or CWNTs alone.

## 1. Introduction

Silver nanoparticles (AgNPs) are one of the most used nanomaterials in our economy because of their various properties of commercial interest [1,2]. They are used in electrical appliances (washing machines), paints, cosmetics, deodorants/soap, plastic food packaging, and clothes because of their antifouling and biocidal properties [3]. It is estimated that the production of AgNPs is between 450 and 500 tons per year [4], which can ultimately contaminate the environment [5]. The ever-increasing marketing of AgNP-containing materials will likely result in the development of more complex nano-devices or nanomaterials. Recently, AgNPs were attached to carbon-walled nanofibers (AgNPs–CWNTs), producing more complex nanomaterials with perhaps inadvertent emergent toxic properties that may not be entirely explained by their individual components. AgNP–CWNT composites are currently used to improve electrical conductivity, enhance tensile strength, hardness and elastic properties of materials for various commercial applications. For example, they are used in electric motors, sensing devices, tear-resistant clothes, and armor. They introduce semi-conductor or conductive films in coatings, plastics, flat screen displays, and electromagnetic wave shielding and lithium battery anodes. These novel applications of AgNP–CWNT composites contribute to increased contamination of aquatic and soil ecosystems through solid waste disposal sites and municipal/industrial wastewaters. However, although the levels of AgNPs in municipal effluents are relatively low, reaching 0.025 µg/L in some effluents [6], it was estimated that AgNPs represented 1.7–7.6% of total suspended Ag in effluents; but the contribution of Ag from nanocomposites, such as AgNPs–CWNTs, is not yet known. A bioconcentration factor of 5 was found in *Daphnia magna* exposed to AgNPs [7], suggesting some potential to bioconcentrate AgNPs during long-term and continuous exposure to municipal effluents.

*Hydra vulgaris* Pallas 1766 has been used as a model organism for medical and environmental studies since the 1950s [8,9,10]. Hydra is the equivalent of anemones family (*Cnidaria* phylum) in freshwater. It is a simple organism composed of a tubular body for digestion and reproduction and a head (mouth) equipped with 7 tentacles (Figure S1). The size of hydra is between 2 mm and 10 mm long with the base usually attached to a solid substrate (branches, stones and vegetation) and the tentacles are used for catching and immobilizing prey for nourishment. Their food consists of small microcrustaceans, such as water fleas and copepods. The hydra is usually unisexual, although some species are hermaphrodites. This organism has remarkable regenerative abilities, continuing to grow and reproduce without significant aging, making it a convenient model for regenerative studies for the medical community. Hydra reproduces by budding forming polyps producing offsprings throughout its life, with doubling time every 4–5 days depending on media composition, temperature, and feeding regime [11]. The sublethal and lethal toxicity of various environmental pollutants, drugs, and effluents manifests by characteristic and successive changes in morphology as the severity (irreversible) of effects increases: tentacle retraction and budding (reversible), loss of tentacles (reversible), contraction of tubular body (tulip), and disintegration (Figure S1). The formation of the tulip and disintegration stages are considered lethal and irreversible, while the first manifestations are reversible. Because of the small size of hydra, sublethal effects investigations at the molecular level are more difficult in environmental toxicological investigations. Quantitative reverse transcription polymerase chain reaction (qRT-PCR) is a well-established highly sensitive and specific methodology to quantify the levels of mRNA transcripts to determine the early molecular effects of xenobiotics. In the attempt to refine this bioassay to investigate the mode of action of chemicals and mixtures, a qRT-PCR methodology was developed to target some of the critical physiological targets for nanomaterials, such as oxidative stress (superoxide dismutase and catalase), protein synthesis (elongation factor 1), protein salvage and autophagy (ubiquitin–proteosome salvage pathway), regeneration and growth (serum response factor 1), and oxidized DNA repair (8-oxoguanine DNA glycosylases) (Table 1). Indeed, nanomaterials were found to increase protein damage, leading to ubiquitinoylation for the proteasome salvage pathway and autophagy in fish and mussels [12,13]. These effects were seemingly specific to nanomaterials, since the dissolved fraction of AgNPs did not produce these effects within the tested concentration range. The toxicity of carbon nano-onions was investigated in hydra and revealed no toxicity at 0.1 mg/L based on morphological changes [14]. Studies on the toxicity of nanomaterial composites in aquatic invertebrates, such as hydra, are lacking at the present time. In addition, this is the first study to consider the toxicity of complex nanomaterials at the gene expression using quantitative reverse transcriptase polymerase chain reaction (qRT-PCR). The development of rapid and sensitive biomarkers at the gene expression level should provide a more detailed understanding of the nanoecotoxicity of complex nanomaterials, such as AgNPs–CWNTs, that precedes changes in morphology in hydra.

The purpose of this study was therefore to examine the toxicity of AgNP–CWNT composites in *Hydra vulgaris* at both the morphological and gene expression levels. Gene expression was analyzed using the very sensitive qRT-PCR gene expression array. Moreover, the toxicity of two composites differing in Ag contents (10 and 50% AgNPs on the CWNT fibers) were examined and compared with AgNPs and CWNTs alone in the attempt to highlight novel emergent toxicity properties in more complex nanomaterials/composites. The null hypothesis statement is that the toxicity of AgNP–CWNT composites could be explained by the levels of AgNPs or CWNTs alone.

## 2. Methods

### 2.1. Sample Preparation

The nanocomposites of silver and carbon nanotubes, and CWNT were purchased at US Research Nanomaterials (Houston, TX, USA). The 20 nm diameter citrate coated AgNPs were purchased from nanoComposix (San Diego, CA, USA). The CWNTs were composed of multiwalled graphene oxide (5–10 nm inside and 55 nm outside diameter) between 10 and 30 µm long. The AgNP–CWNT composites consisted of 10% AgNPs and 90% CWNTs and 50% AgNPs and 50% CWNTs, by weight. The samples were purchased as 3% water dispersion and diluted to 1 mg/mL in 1 mM CaCl_2_ containing 0.4 mM tris aminoethane sulfonate (TES) buffer pH 7.4 (hydra medium). Stock solutions were characterized by transmission electron microscopy (TEM) and energy dispersive X-ray spectroscopy (Centre for characterization and microscopy of materials (CM), (Polytechnique Montréal, Montréal, QC, Canada). TEM images were analyzed using the image software ImageJ (version 1.1) [15].

### 2.2. Hydra Toxicity Assessments

The assays were performed using standardized methodology for the cnidarian *Hydra vulgaris* and *attenuata* [16]. The hydras were cultivated in crystallization bowls containing 200 mL of hydra media. They were fed with fresh suspensions of *Artemia salina* each day, as previously described, giving a doubling time of 4–5 days [17]. For the exposure step, adult hydra (3 individuals per 4 mL in 24 well microplates with *N* = 3 per concentration) were plated in the hydra medium. They were exposed for 96 h at 20 °C to increasing concentrations of two AgNP–CWNT composites as 0, 1.56, 3, 6, 12, 25, 50, and 100 µg/L as total Ag added corresponding to 1.56 to 100 µg/L of CWNTs for the 50% AgNP–CWNT composite and to 14–900 µg/L CWNTs for the 10% AgNPs–90% CWNT composite. The 96-h exposure time was selected based on preliminary experiments that the maximum effects for morphological changes occurred and stabilized (lower variation) after 72 h of exposure time. For AgNPs alone, the hydra were exposed to the same concentration range of Ag for the nanocomposites and for CWNTs to 27, 56, 112, 225, and 450 µg/L. No signs of precipitation were observed visually for the CWNTs and, using the reported absorption at 480 nm, for AgNPs and AgNP–CWNT composite [18]. Following 96-h exposure, the lethal and sublethal effects were determined using a 4–6 X stereomicroscope. The 96 h hydra bioassay determines the lethal (the lethal concentration that kills 50% of the hydra-LC50) based on severe morphological changes, such as loss of antenna/tentacles and severe contraction of the tube-like body reminiscent of a tulip flower (Figure S1). These changes are considered irreversible since the organisms do not recuperate and lead to mortality. The sublethal toxicity determines the effect concentration where 50% of the hydra (EC50) exhibit the following reversible morphological changes: tentacle shortening with bud formation at the head. The hydra was not fed during the exposure time. Given that the exposure time encompasses the reproduction period (appearance and release of polyps) of 4–5 days, this assay could be considered chronic. The surviving hydra (with no morphological alterations) were resuspended in RNAlater solution and stored at −20 °C for subsequent gene expression analysis.

### 2.3. Gene Expression Analysis

Total RNA was extracted from *N* = 6 hydra using the RNeasy Plus Mini Kit (Qiagen, Toronto, ON, Canada). RNA concentration and purity were assessed at 260 and 280 nm with the NanoDrop 1000 (Thermo Fisher Scientific, Mississauga, ON, Canada), and RNA integrity was verified using the TapeStation 4150 System (Agilent) with the Agilent RNA ScreenTape Instrument (cat # 5067-5576, Agilent Technologies Inc., Santa Clara, CA, USA). Reverse transcription was performed with the QuantiTect^®^ Reverse Transcription Kit (Qiagen), ensuring the complete removal of genomic DNA, following the manufacturer’s instructions. The resulting cDNA samples were stored at −80 °C for quantitative real-time RTPCR analysis.

All qPCR determinations used the following commercial kit, SsoFast™ EvaGreen^®^ Supermix (Bio-Rad, Mississauga, ON, Canada) and the CFX96 Touch Real-Time PCR Detection System (Bio-Rad, Mississauga, ON, Canada). For each selected primer pair (Table 1), a calibration curve (starting cDNA concentration: 10 ng, 8 serial dilutions) was generated, with PCR efficiency values ranging between 95% and 115%, and the limit of quantification was determined. Each reaction was run in duplicate and consisted of 5 µL cDNA, 6.5 µL of 2× SsoFast EvaGreen Supermix (Bio-Rad), 300 nM of each primer, and DEPC-treated water (Thermofisher, Ottawa, ON, Canada) up to a total volume of 13 µL. Cycling parameters were as follows: 95 °C for 30 s, then 40 cycles of 95 °C for 5 s, and 60 °C for 10 s, for HPRT, RPLPO, EF1, DDC1, SRF, and OGG; 95 °C for 30 s, then 40 cycles of 95 °C for 5 s, and 56 °C for 10 s, for CAT and MANF; and 95 °C for 30 s, then 40 cycles of 95 °C for 5 s, and 56 °C for 30 s, for MAPCI3. Two housekeeping genes were used for normalization [19] with the following genes: hypoxanthine-guanine phosphoribosyltransferase (HPRT) and 60S acidic ribosomal protein P0-like RPLPO. Amplification specificity was verified using a melting curve profile analysis. A no-template control (NTC) was included on each plate. Gene expression analysis was performed using CFX Maestro (Bio-Rad).

### 2.4. Data Analysis

The 96 h LC50 and EC50 obtained from each the bioassay in hydra, were determined using the Spearman–Karber method [20]. Toxicogenomic data were normalized against control hydra and were reported as effect thresholds (ET) as defined: threshold = (no effect concentration x lowest significant effect concentration)^1/2^ in µg/L. The gene expression data were submitted to an analysis of variance following Levene’s test for normality and variance homogeneity. In the case that the data was non-parametric, they were log transformed. Critical difference from the controls was determined using the Fisher least square difference test. An analysis of covariance between the exposure concentrations (for SRF1) and a given gene expression data as the covariate (CAT or EL1) to remove the influence of covariates on the concentration–response curves. Relationships between toxicity (LC50 and EC50) and gene expression data (ET) were determined using the Pearson moment correlation test. The biomarker data were also analyzed by hierarchical tree to determine similarity of effects between the nanocomposites, AgNPs and CWNTs using the squared correlation coefficient (1 − r^2^ or 1 − R) as the metric distance. iscriminant function analysis was also determined to identify key gene expression changes that best discriminate the two AgNP–CWNT composites, AgNPs, CWNTs, and the control hydra. Significance was set at *p* < 0.05. All the statistical analyses were conducted using SYSTAT (version 13.2, USA).

## 3. Results

The CWNTs, AgNPs, and their composites were examined by TEM analysis (Table 2). Citrate coated AgNPs (20 nm diameter) and CWNTs were included as a proxy for the AgNP–CWNT composites from nanoComposix (San Diego, CA, USA). In the stock solutions, AgNPs were mostly generally monodispersed with some aggregation. The aggregates were relatively stable in water, since the total measured concentrations corresponded to 80–105% of the nominal concentration in aquarium water, based on spectrometric analysis of 1 mg/L stock solution after one hour. This suggests that hydra was mostly exposed to the nanoparticulate form of AgNPs in this study, rather than the dissolved Ag. For the carbon-walled nanotubes (CWNTs), 10–30 µm long fibers were generally well dispersed (not aggregated) with an inside diameter of approximately 8–10 nm and an outside diameter of 50–60 nm for the multiwall or carbon sheets. The CWNT suspensions were surprisingly stable, as no evidence of precipitation was found over one week at 20 °C. For the 10% AgNPs–90% CWNT composites, AgNPs were attached at the surface of the CWNTs as monomers, covered with a thin carbon film. Non-attached AgNPs were also found and were mostly in aggregated state. For the 50% AgNPs–50% CWNTs, the same pattern was observed. Based on image analysis, it was estimated that 30–40% of the AgNPs were attached to the carbon fibers, and the remaining fraction was present as AgNP aggregates.

**Figure 1:**
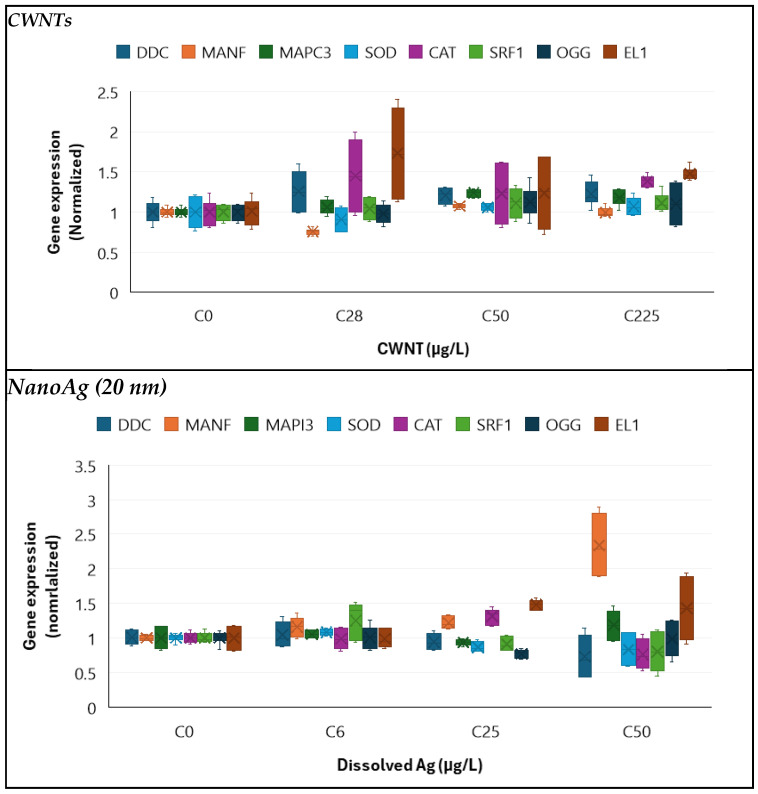

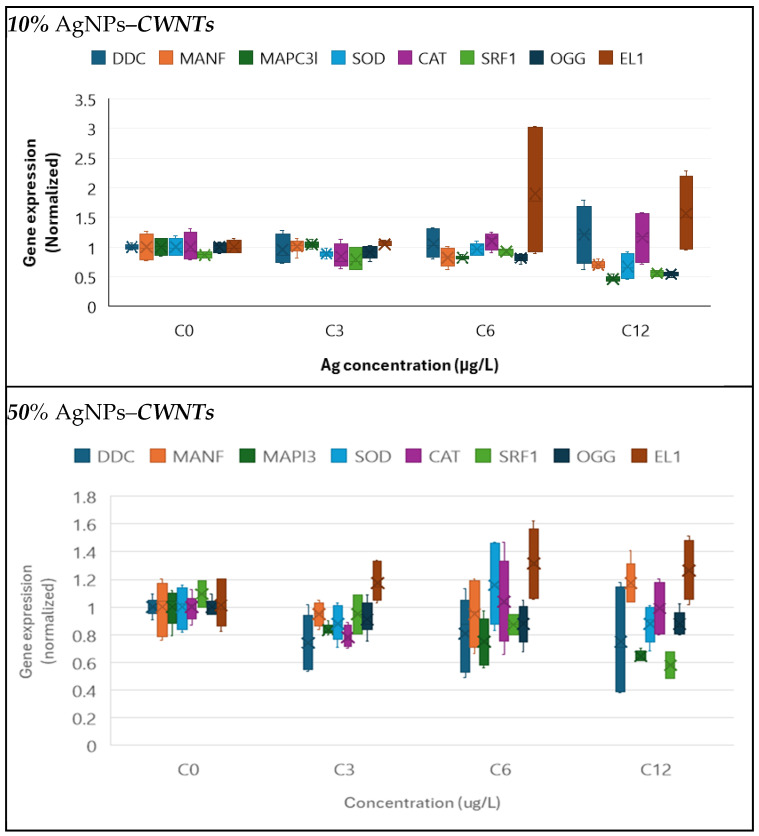
Gene expression changes in hydra exposed to various forms of silver. Hydra were exposed to carbon-walled nanotubes (CWNTs), AgNPs, 10% AgNPs–90% CWNT, and 50% AgNPs–CWNT composites. The data is expressed as the median (star symbol), the 25th–75th quantiles (box), and the data range (minimum–maximum, brackets).

The toxicity and changes in gene expression were determined in hydra for the various states of AgNPs and CWNTs (Figure 1). For CWNTs, the exposure concentration ranged from 28 to 225 µg/L with no appearance of any signs of toxicity based on morphological changes (Table 3). Exposure to CWNTs generally increased gene expression, with the exception of MANF showing non-monotonic decrease gene expression at the lowest exposure concentration (28 µg/L) only to return to control levels for the higher concentrations (50–225 µg/L). Aside from MANF, SOD, and SRF1, most gene expression was significantly increased at the threshold concentration of 105 µg/L. For citrate-coated AgNPs, only sublethal effects (EC50) based on reversible morphological changes were observed, with an EC50 of 9 µg/L. The following genes were increased by this form of silver at a threshold concentration of 12 µg/L: MANF, CAT, and EL1. SOD and OGG gene expression were significantly suppressed, while no effect on SRF1 gene expression was observed. For the 10% AgNPs–90% CWNT composite, the LC50 was observed at 30.5 µg/L and EC50 at 8.2 µg/L as Ag, or 2600 µg/L LC50 and 74 µg/L EC50 as CWNTs. DDC and CAT transcripts (mRNA) were significantly induced at a threshold concentration of 8.5 µg/L Ag (76.5 µg/L as CWNTs), while EL1 was induced at a threshold concentration of 4.2 µg/L Ag (or 38 µg/L CWNTs). Decreased gene expression was found for MANF (4.2 µg/L as Ag or 38 µg/L as CWNTs), MAPC3l (4 µg/L as Ag), OGG (4.2 µg/L as Ag), and SOD (8.5 µg/L as Ag). SRF1 transcript levels were significantly decreased at 3 µg/L only after covariance analysis with either CAT or EL1 gene expression as the covariate. For the 10% AgNPs–90% CWNT composite, DDC was significantly correlated with CAT (r = 0.83), SRF1 (r = 0.71), and EL1 (r = 0.67). SOD gene expression was correlated with OGG (r = 0.74) and MANF (r = 0.60). CAT transcript levels were significantly correlated with EL1 (r = 0.62). EL1 transcripts were correlated with MANF (r = −0.51) and SRF1 (r = 0.49).

For the 50% AgNP–50% CWNT composite, the lethal (LC50) and sublethal (EC50) concentrations were 78 µg/L and 8.5 µg/L as silver (corresponding to the same CWNT concentrations). MANF and EL1 were significantly induced at a threshold concentration of <3 and 85 µg/L as Ag and CWNTs, respectively. MAPC3l, SOD, and OGG were significantly decreased at a threshold concentration of 8.5 µg/L as both Ag and CWNTs. CAT gene expression was significantly reduced at the lowest exposure concentration of 3 µg/L. SRF1 transcript levels were significantly decreased at 12 µg/L only after covariance analysis with either CAT or EL1 gene expression as the covariate. Correlation analysis revealed DDC gene expression was correlated with EL1 (r = −0.59). SOD gene expression was significantly correlated with MANF (r = 0.72), CAT (r = 0.87), SRF1 (r = 0.77) and EL1 (r = 0.74). CAT transcription was significantly correlated with SRF1 (r = 0.59), MANF (r = 0.7) and EL1 (r = 0.73). El1 gene expression was correlated with MANF (r = 0.81).

**Figure 2:**
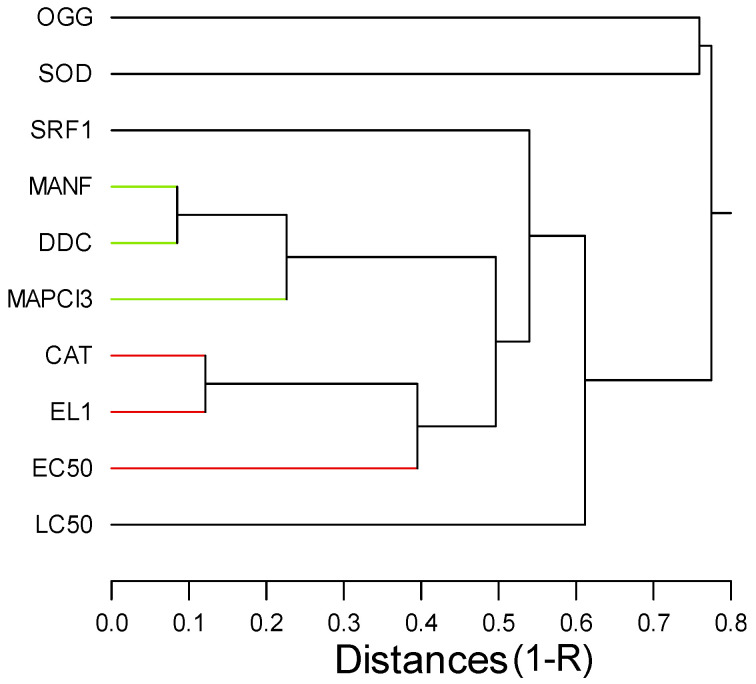
Hierarchical tree analysis of CNT, various forms of silver, and toxicity. The analysis was performed on the toxicity thresholds for gene expression changes (Table 2). The distance was calculated based on (1 − R) on the x axis.

**Figure 3:**
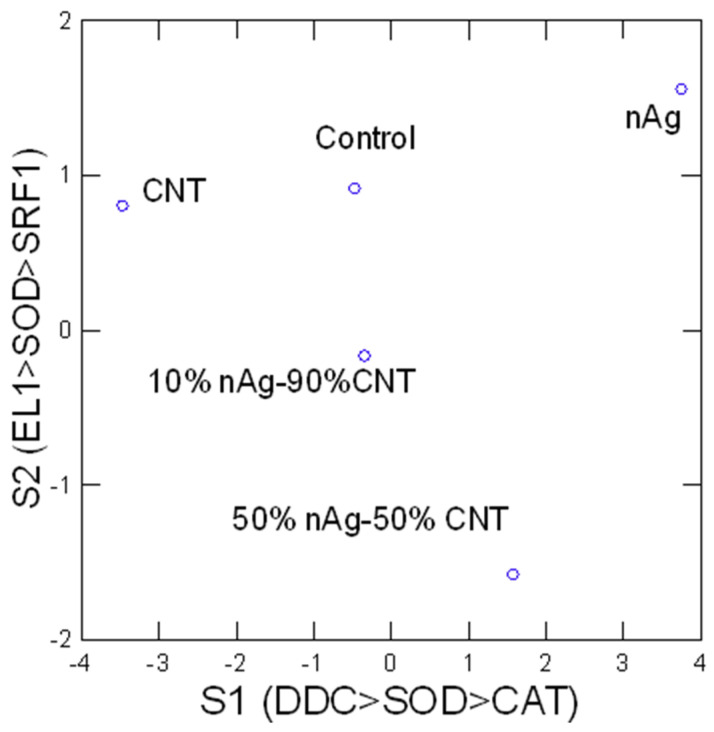
Discriminant function analysis of gene expression. Discriminant function analysis was performed on the gene expression data at the same concentration range (3–6 ug/L. The points represent the mean distribution of the scored data and the most significant gene changes are found in the parenthesis for each factor (axis).

In an attempt to gain a global understanding of the toxic effects of AgNP–CWNT composites, a hierarchical and discriminant function analysis was performed. A hierarchical tree analysis was performed between the gene expression thresholds and lethal/sublethal toxicity data (Figure 2). The analysis revealed that the lethal toxicity (LC50) was not related to changes in gene expression, contrarily to the sublethal toxicity (EC50), which was significantly related to gene expression changes for CAT and EL1 cluster (i.e., EL1 and CAT were significantly related with each other). Moreover, DDC, MANF, MAPC3l, and SOD transcript levels formed a cluster with significant associations with each other, but a weaker link with the EC50. The strong correlation between DDC and MANF gene expressions suggest that dopamine signaling was associated to dopamine neuron repair. The concentration-response data for the eight genes were examined using a discriminant function analysis to determine the similarities in the toxicity responses among CWNT, pure AgNPs, and AgNP–CWNT composites (Figure 3). The analysis revealed that the effects of pure AgNPs and CWNTs were mostly discriminated by the x axis with the following gene expression changes (DDC > SOD > CAT), which are mainly involved in oxidative stress and neuroactivity (Dopa decarboxylase). For the AgNP–CWNT composites, the responses differed from single AgNPs and CWNTs by the second component on the y axis with the following gene expression changes (EL1 > SOD > SRF1), suggesting changes in protein synthesis activity, oxidative stress, and regeneration. Although SRF1 changes were generally decreased by the exposure concentrations, CAT and protein synthesis (EL1) were increased for the 50% AgNP–CWNT composite. The same results were also observed for the 10% AgNP–CWNT composite, suggesting that SRF-1 signaling was not only related to exposure concentration of AgNP–CWNT components, but to oxidative stress and protein synthesis as well.

## 4. Discussion

The lethal toxicity (LC50) of the tested compounds were 450 µg/L for AgNPs and CWNTs, at 30.5 µg/L for 10% AgNPs–90% CWNTs and 78 µg/L for 50% AgNPs–50% CWNTs. The EC50s were more closely related to those needed for gene expression changes, especially for the AgNP–CWNT composites. In a previous study, the LC50 of single-walled CWNTs were between 1 and 10 mg/L, corroborating the present findings [21]. In another study, the toxicity of CWNTs coated with humic acid were above 10 mg/L in hydra [22], suggesting that the observed toxicity in the present study mainly comes from the AgNPs and AgNP–CWNT composites. In respect to AgNPs (20 nm diameter), the LC50 and EC50 were >100 and 9 µg/L, respectively; whereas, for larger citrate coated AgNPs (50 nm), the LC50 and EC50 were 56 and 22 µg/L, suggesting some slight differences albeit at the same order of magnitude [23]. However, the toxicity of free Ag^+^ ions was found to be 4 µg/L for the LC50 and 2.6 µg/L for the EC50, showing more lethal effects than the other tested compounds, but close to the EC50 values for the AgNP–CWNT composites. The lower toxicity of citrate coated AgNPs in the present study could be explained by the negatively charged citrate coating of the nanoparticles, diminishing bioavailability to hydra, thus reducing toxicity compared to the free Ag^+^. The sublethal toxicity (EC50) was closely associated to EL1 and CAT gene expression, suggesting impacts at the oxidative stress and protein synthesis levels. Changes in gene expression occurred at threshold concentrations sometimes below 3 µg/L, before the appearance of morphological changes. This was also observed following discriminant function analysis, where genes involved in oxidative stress (SOD, CAT), protein synthesis (EL1), dopamine neural activity (DDC) and regeneration (SRF1) were the genes that best discriminated the toxic effects of the AgNP–CWNT composites, AgNPs, and CWNTs. In adult hydra, the abundance of SRF transcripts varies during the day following a circadian cycle [24]. The pacemaker of this diurnal rhythm is the feeding regime. Expression of SRF1 occurs in the ectodermal layer, in the endodermal epithelial cell mass, and in I-cells of the body. Its expression ceases when I-cells differentiate into nerve, nematocytes, or gland cells. This suggests that either the feeding regime or cell regeneration and differentiation is decreased in hydra exposed to the AgNP–CWNT composites. On the one hand, exposure to large microplastics (<400 µm) within the size range of the tube length of the CWNTs (10–30 µm) were found to reduce feeding rates in hydra [25]. On the other hand, exposure to small polymethylmethacrylate plastic nanoparticles (40 nm) had no effect on hydra feeding activity, albeit at relatively high concentrations of 10–40 mg/L [26]. Decreased algal feeding activity was also observed in *Daphnia magna* exposed to 3.9 mg/L CWNTs after 48 h with fiber length > 1 µm, based on clay particle size effects on algal feeding [27]. This suggests that CWNT fibers length in the microplastic scales could also have an impact on feeding rates and perhaps towards SRF1 gene expression.

It was previously found that the appearance of rod-like nanocrystals stimulated neural activity in hydra [28]. Indeed, exposure to quantum rods, but not spheres, induced an unexpected Ca^2+^-dependent tentacle-writhing behavior that relies on tentacle neurons. Dopamine signaling is involved in awake/activity periods in hydra and tentacular regeneration [29,30]. Flatworms and hydra exposed to neurotransmitters involved in wakefulness in vertebrates (acetylcholine, dopamine, glutamate, and histamine) increased activity time. Conversely, sleep inducing neurotransmitters (adenosine, GABA, and serotonin) increased resting times. Exposure to CWNTs induced DDC gene expression (involved in the conversion of dopamine from L-DOPA), while this effect was lost with the AgNP–CWNT composites. However, gene expression involved in regeneration (SRF1) was significantly decreased after correction with either CAT or EL1 gene expression in hydra exposed to the AgNP–CWNT composites (ANCOVA *p* < 0.01 for concentration and CAT or EL1 as the covariate), where SRF1 gene expression was correlated with dopamine production (DDC), oxidative stress (SOD, CAT), and protein synthesis (EL1). This suggests that the active state (feeding) of hydra is stimulated to these nanocomposites and involves the production of oxidative stress at the expense of regeneration, since SRF1 expression was significantly reduced. This could represent the emerging toxicity of the nanocomposites because these effects were not observed with CWNTs and AgNPs alone. N-arachidonoyl dopamine accelerated tentacle formation in both the gastral and basal fragment of the body [30]. These results are in line with a previous study showing that dopamine synthesis inhibitors and antagonists decreased hydra regeneration and growth [31]. The correlation between DDC and MANF gene expressions suggests that dopamine signaling was associated to cell damage (dopamine acts as a pro-oxidant) [32]. Analysis of covariance (exposure concentration as the treatment and DDC as the covariate) of dopamine cell damage revealed that cell damage was not solely explained by DDC gene expression involved in the production of dopamine. Hence, the observed changes in repair of dopaminergic neurons (MANF) were not associated to reactive oxygen species during dopamine metabolism (DDC). These results suggests that toxic stress occurs at concentrations well below the appearance of morphological changes giving rise to the possibility of harmful effects for longer periods than the 96-h period of exposure used in this study. More research should be performed for longer periods in hydra over many doubling times (doubling time of 4.5 days). This would be a more realistic scenario, since the hydra would be exposed over the long-term in polluted environments by these composites, as well as considering that their food source could be contaminated as well.

## 5. Conclusions

The toxicity of AgNP–CWNT composites were shown to induce sublethal morphological changes at concentration below those for AgNPs and CWNTs alone, suggesting that the tube-like shape of AgNPs-CWNT could induce sub-lethal toxicity in hydra. Sublethal effects were associated to oxidative stress (CAT) and protein synthesis (EL1), while lethality involved a broader subset of genes, such as dopamine activity (DDC), MAPC3l, and MANF. SRF1 gene expression was highly correlated with oxidative stress biomarkers and dopamine signaling, and its expression readily decreased following covariance analysis. The data suggests that AgNP–CWNT composites introduce changes at the gene expression levels and morphological changes that are not solely explained by AgNPs or CWNTs alone. Further investigations on the release and fate of AgNP–CWNT composites in aquatic ecosystems are thus mandated.

## Figures and Tables

**Table 1:** Transcription gene identity and sequences.

Function		Gene Name	Forward/Reverse (5′---->3′)	Amplicon (bp)
Housekeeping genes		hypoxanthine-guanine phosphoribosyltransferase-like **HPRT**	GAA TTG AAC GCA TGG CTC GT/ GTC TTG GCT GAA CCG AAA ACC	98
		60S acidic ribosomal protein P0-like **Rplp0-1**	CTG AGG CTG CTC TTC TTG CT/ GGA CTG AAA ATG CTT CCG TTG T	94
Autophagy and Ub-pathway		microtubule-associated protein 1 chain 3 light) **MAPC3l**	CCA GAG AAA GCG AGA ATC CGA/ TGG AGA GCA TAC CAA CTG TCA T	152
Neuroactivity (repair of dopaminergic neurons)		Mesencephalic astrocyte-derived neurotrophic factor homolog **MANF**	CCA CTC GCA TAC TAC AAG CCT/ ACA ACC ACT ACA AGT CTC ACC C	180
Antioxidant response		superoxide dismutase [Cu-Zn]-like **SOD**	ACC TGG TAA GCA CGG TTT TCA/ TGC ACC ACT CCA TCT TTA CCA	171
		catalase-like **CAT**	ACA GCC TCA ATG ACT GTT GGG/ CCA CTC CAT TCA GAG CAG CC	196
DNA damage and repair		8-Oxoguanine DNA Glycosylase **OGG**	TGT GAC TGG AGT TGA AGA TGC T/ ACT CCA GGC AAT GAG CAA AGA	174
Regeneration and Stem factor		Serum Response Factor **SRF1**	CTT GTG GCA TCG GAA ACA GG/ TGC TTT GCC ACT TTC AGA GGT A	84
	Neural activity	Dopa Decarboxylase **DDC**	GCC CCA GTT GAG CCA GAT AA/CAG TGA GTG ACA CCT GGC AT	77
	Protein synthesis	elongation factor-1 alpha **EL1**	TGC TCC TGG ACA TCG TGA CT/ CAA CGA TGA GTA CGG CAC AAT C	77

**Table 2:** Physicochemical characteristics of the nanosilver composites.

Silver Form	AgNPs Diameter (nm)	CNT Diameter-Fibre Length	TEM	General Features (TEM Observation)
AgNPs citrate coated	20 ± 3 nm	--	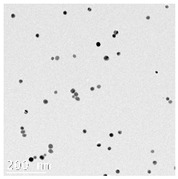	Monomeric aspect. No evidence of aggregation.
CWNTs	---	8 nm (inside) 55 nm (outside) 10–30 µm	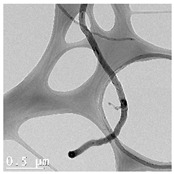	Well dispersed fibers with no aggregation or coalescence.
10% AgNPs–90% CWNTs	24 ± 4 nm	8 nm (inside) 10–30 µm	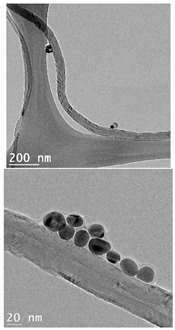	Well dispersed fibers with AgNPs bound to carbon fibers. The bound AgNPs appears encapsulated by a thin carbon sheet. About 30% of AgNPs is attached to CWNTs.
50% AgNPs–50% CWNTs	23 ± 3 nm	8 nm (inside) 10–30 µm	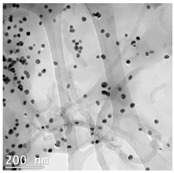	Well dispersed fibers with higher AgNPs density attached to carbon fibers. The bound AgNPs appears encapsulated by a thin carbon sheet. About 40% of AgNPs is attached to CWNTs.

**Table 3:** Toxicity and gene expression changes in hydra exposed to different concentrations of nanocomposites of silver and carbon nanotubes.

Elements	CWNT (µg/L)	nAg (µg/L)	10% nAg-90%CNT (Ag; CNT µg/L)	50% nAg-50%CNT (µg/L)
DDC	<28 (+)	35 ^1^ (-)	ND ^2^	<3 (-)
MANF	<28 (-)	35 (+)	8.5; 76.5 (-)	8.5 (+)
MAPCI3	37 (+)	35 (-)	4.2; 38 (-)	<3 (-)
SOD	ND	35 (-)	8.5; 76.5 (-)	ND
CAT	<28 (+)	12 (+)	ND	<3 (-)
SRF1	ND	ND	<3 (-)	8.5 (-)
OGG	ND	12 (-)	ND	8.5 (-)
EL1	<28 (+)	12 (+)	4.2; 38 (+)	4.2 (+)
Hydra				
LC50	>450	>100	30.5 (22–41)	78 (67–92)
EC50	>450	9 (6–13) ^3^	8.2 (7–9.5)	8.5 (5.7–12.6)
# genes affected	5/8	7/8	4/8	6/8

^1^ calculated based on the threshold concentration: (Lowest significant effect concentration × No effect concentration)^1/2^. ^2^ ND: no effect detected. ^3^ 95% confidence interval.

## Data Availability

Data is contained within the article.

## Supplementary Materials

The following supporting information can be downloaded at: https://www.mdpi.com/article/10.3390/nano14231955/s1, Figure S1: Characteristic morphological changes in *Hydra vulgaris* during toxicity. Normal A, tentacle contraction and budding B, severe contraction C; tulip stage D and disintegrated E.
