# Supplementary material for: Lethal and Sublethal Toxicity of Nanosilver and Carbon Nanotube Composites to Hydra vulgaris—A Toxicogenomic Approach
Source: Nanomaterials (Basel). 2024 Dec 5;14(23):1955. doi: 10.3390/nano14231955 (PMC11643480; doi:10.3390/nano14231955)
Supplement: Supplementary file 1 [file nanomaterials-14-01955-s001.zip › nanomaterials-3341723-supplementary.pdf]

Supplementary material

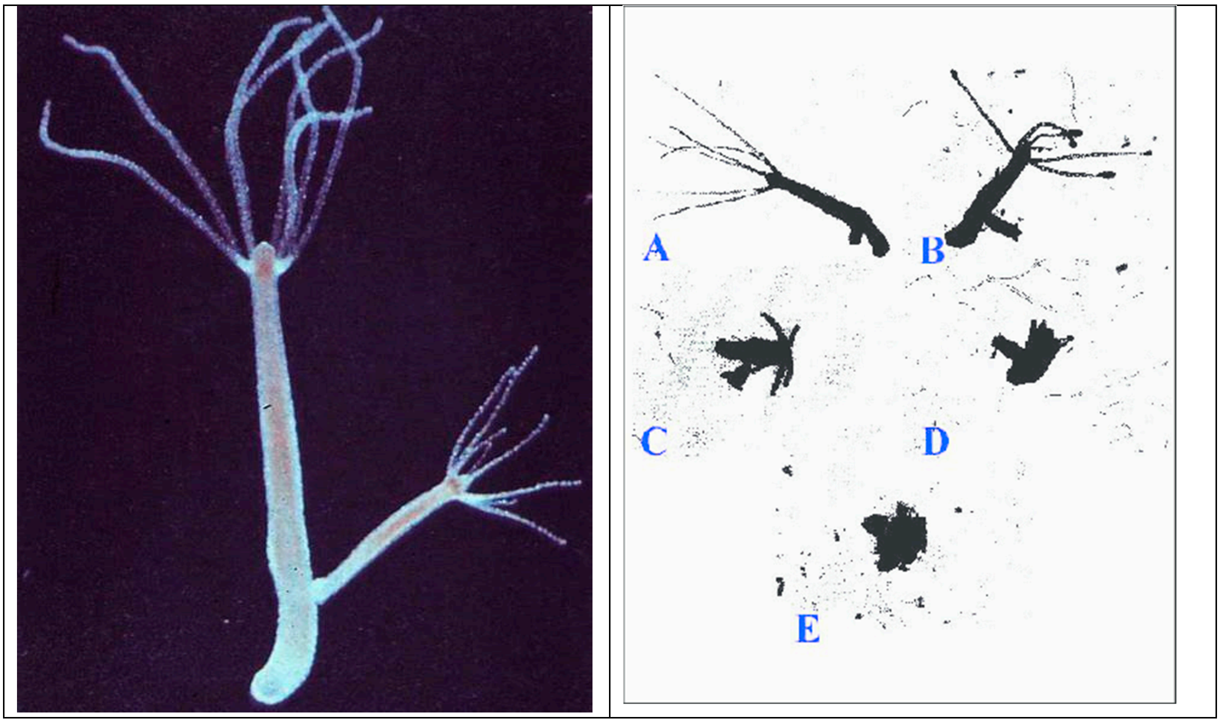

Figure S1. Characteristic morphological changes in *Hydra vulgaris* during toxicity.
Normal A, tentacle contraction and budding B, severe contraction C; tulip stage D and desintegrated E.
